# Molecular integration of the anti-tropomyosin compound ATM-3507 into the coiled coil overlap region of the cancer-associated Tpm3.1

**DOI:** 10.1038/s41598-019-47592-9

**Published:** 2019-08-02

**Authors:** Miro Janco, Michael J. Rynkiewicz, Liang Li, Jeff Hook, Eleanor Eiffe, Anita Ghosh, Till Böcking, William J. Lehman, Edna C. Hardeman, Peter W. Gunning

**Affiliations:** 1 School of Medical Sciences, University of New South Wales Sydney, Sydney, NSW 2052 Australia; 2 Department of Physiology & Biophysics, Boston University School of Medicine, 72 East Concord Street, Boston, MA 02118 USA; 3 Single Molecule Science and ARC Centre of Excellence in Advanced Molecular Imaging, University of New South Wales Sydney, Sydney, NSW 2052 Australia

**Keywords:** Cancer, Structure-based drug design, Cytoskeletal proteins

## Abstract

Tropomyosins (Tpm) determine the functional capacity of actin filaments in an isoform-specific manner. The primary isoform in cancer cells is Tpm3.1 and compounds that target Tpm3.1 show promising results as anti-cancer agents both *in vivo* and *in vitro*. We have determined the molecular mechanism of interaction of the lead compound ATM-3507 with Tpm3.1-containing actin filaments. When present during co-polymerization of Tpm3.1 with actin, ^3^H-ATM-3507 is incorporated into the filaments and saturates at approximately one molecule per Tpm3.1 dimer and with an apparent binding affinity of approximately 2 µM. In contrast, ^3^H-ATM-3507 is poorly incorporated into preformed Tpm3.1/actin co-polymers. CD spectroscopy and thermal melts using Tpm3.1 peptides containing the C-terminus, the N-terminus, and a combination of the two forming the overlap junction at the interface of adjacent Tpm3.1 dimers, show that ATM-3507 shifts the melting temperature of the C-terminus and the overlap junction, but not the N-terminus. Molecular dynamic simulation (MDS) analysis predicts that ATM-3507 integrates into the 4-helix coiled coil overlap junction and in doing so, likely changes the lateral movement of Tpm3.1 across the actin surface resulting in an alteration of filament interactions with actin binding proteins and myosin motors, consistent with the cellular impact of ATM-3507.

## Introduction

Tpms are coiled coil dimers that form co-polymers with actin such that the Tpm polymers run along both sides of the actin filament^1–3^. In striated muscle all actin filaments are saturated with Tpm^4,5^; whereas, in the non-muscle cytoskeleton the level of saturation of actin by Tpm can vary from 30–90% depending on the cell type^6^. The stability of the Tpm polymer is dependent on the 4-helix overlap junction between adjacent dimers^7,8^. The importance of this junction is highlighted by the fact that the greatest peptide divergence between Tpm isoforms lies in the N- and C-termini which generate the overlap junction^9^. This is also further highlighted by the finding that the Tpms primarily form homopolymers and regulate the functional capacity of actin filaments in an isoform-specific manner^10^. It is therefore to be expected that the overlap junction contributes substantially to isoform-specific function.

**Figure 1:**
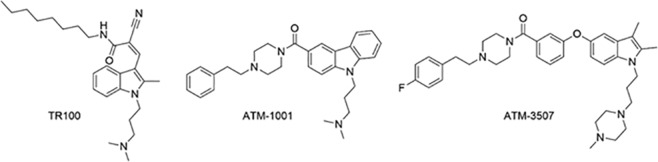
Structures of compounds designed to target Tpm3.1.

Cancer is associated with a profound change in the organization of the actin cytoskeleton and this is accompanied by extensive changes in the levels of the different Tpm isoforms^11–14^. In all cancers studied, only one Tpm isoform, Tpm3.1, is retained at a high level if not increased^15,16^ and accounts for up to 70% of total Tpm in transformed cells^6^. We have therefore developed compounds that target Tpm3.1 as potential anti-cancer agents^15,17^. Both the initial compound, TR100, and the improved compounds ATM-1001 and ATM-3507 (Fig. 1) have significant anti-cancer activity and synergize with anti-microtubule drugs^15,17^.

Anti-Tpm3.1 compounds (ATMs) are designed to bind to the C-terminus of the Tpm3.1 coiled coil and have activity against Tpm3.1 function in *vitro* and *in vivo*. Tpm3.1 protects actin filaments from dilution-induced depolymerization *in vitro* and this is abolished by exposure to ATMs during polymerization^15,17^. As evidence for drug specificity *in vivo*, ATMs inhibit Tpm3.1-mediated regulation of blood glucose levels (via Tpm3.1’s role in inserting the glucose transporter, GLUT4, into the plasma membrane) in wild type, but not Tpm3.1 knock out mice^18^. To date, all studies of ATMs have been limited to *in vitro* and *in vivo* functional assays.

In this study, we have directly measured the association of ATM-3507 with Tpm3.1-containing actin filaments and the impact of ATM-3507 on the physical properties of Tpm3.1 peptide fragments using CD spectroscopy and thermal melts. Our results support the initial binding of ATM-3507 to the C-terminus of Tpm3.1 that subsequently delivers the compound into the overlap junction during the co-polymerization of Tpm3.1 with actin. Molecular dynamic simulation (MDS) predicts that ATM-3507 integrates into the 4-helix overlap domain, but that this can only happen during polymerization. MDS further predicts that integration of ATM-3507 is likely to inhibit lateral movement of Tpm3.1 across the surface of actin.

## Results

### CD spectroscopy and thermal stability of Tpm3.1 peptides in the presence of ATM-3507

**Figure 2:**
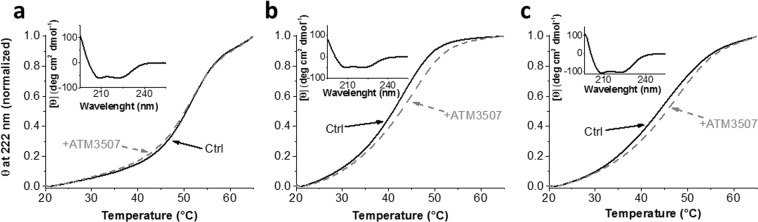
ATM-3507 binds specifically to the C-terminus of Tpm3.1 and the overlap junction. (**a**) Normalized unfolding profile of N-terminal 82AA_Tpm3.1 construct (20 µM) in the absence (black solid line; n = 3) and the presence (gray dashed line; n = 3) of ATM-3507 (100 µM). (**b**,**c**) Normalized unfolding profiles of the C-terminal 109AA_Tpm3.1 construct (20 µM) and the mixture of N- and C-terminal Tpm3.1 constructs (both at 20 µM), respectively. Black solid lines represent controls in the presence of 1% acetonitrile [(**b**) n = 3; (**c**) n = 2] and gray dashed lines are protein samples (20 µM) containing 100 µM of ATM-3507 [(**b**) n = 6; (**c**) n = 6]. The CD spectra of all constructs in the inset of individual panels show the expected α-helical protein profile and were measured at 37 °C prior to temperature ramping experiments. Buffer conditions for both spectra scan and thermal unfolding were 10 mM NaH_2_ PO_4_ pH 7, 150 mM NaCl, 75 µM TCEP, 1% (v/v) acetonitrile.

Peptides corresponding to the C-terminus (residues 139-248) and N-terminus (residues 2–80 with an initial Ala-Gly dipeptide to mimic N-terminal acetylation) of Tpm3.1 either alone or in combination to form the overlap junction produced CD spectra characteristic of the expected α-helical protein profile (Fig. 2 insets in a, b, c). Temperature ramping experiments were then performed in the presence and absence of ATM-3507. The N-terminal peptide showed a characteristic melting profile with no detectable difference in the presence or absence of ATM-3507 (Fig. 2a). In contrast, both the C-terminal fragment (Fig. 2b) and the combination of N- plus C-terminal peptides (Fig. 2c) showed an increase in thermal stability in the presence of ATM-3507. This suggests that ATM-3507 is able to increase the stability of the C-terminus either alone or when it is allowed to interact with the N-terminus.

**Figure 3:**
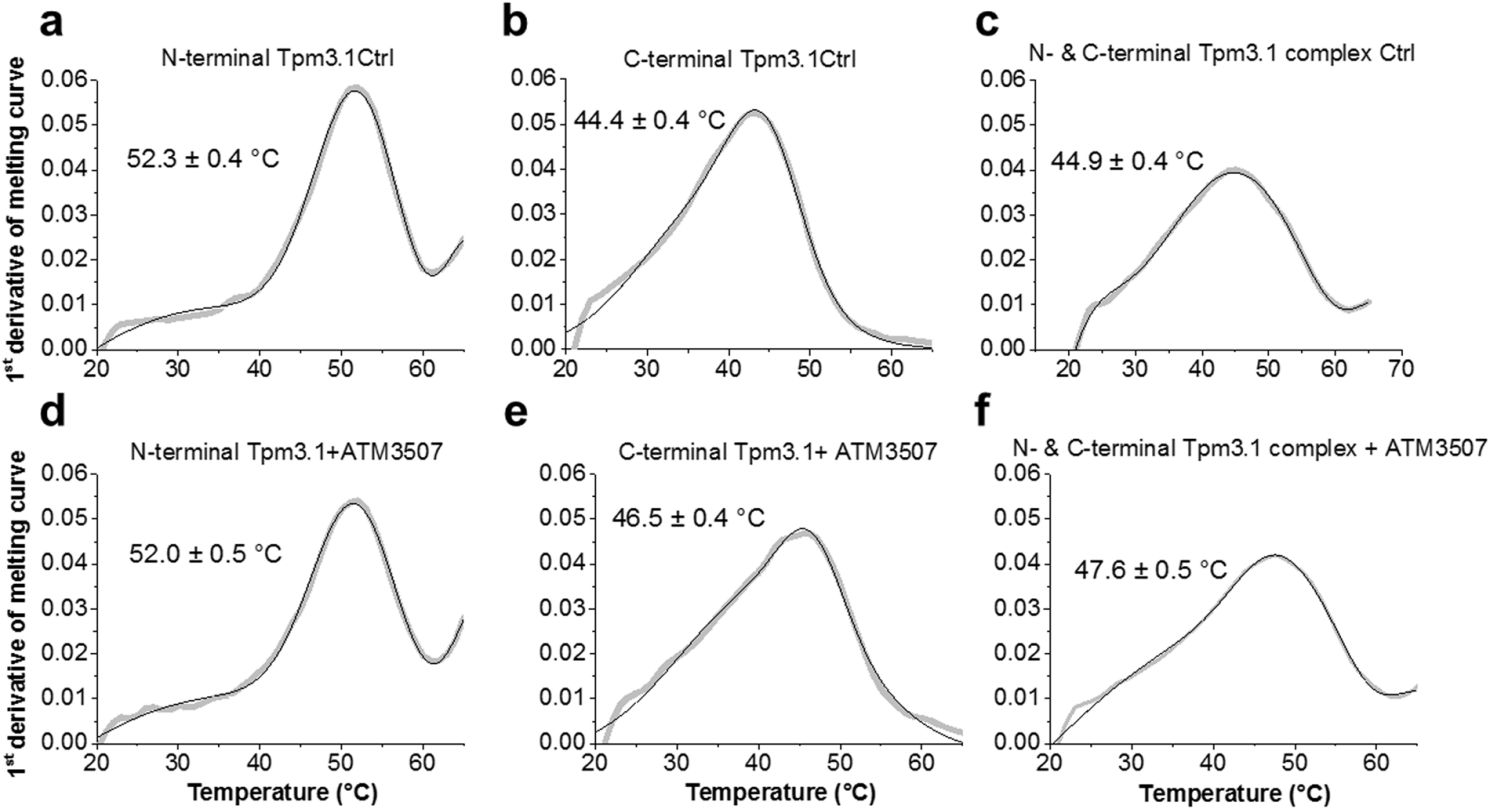
ATM-3507 increases the thermal stability of the C-terminal of Tpm3.1 and the overlap junction. The first derivative of the N-terminal, C-terminal and the overlap junction of Tpm3.1 thermal unfolding data measured by CD in the absence (panels (a–c), respectively) and in the presence (panels d–f, respectively) of 100 µM of ATM-3507. The first derivatives of the data shown in Fig. 2 (gray line) were fitted to multiple Gaussian peaks, smoothed (Savitzky-Golay method; 5 points of window) and differentiated with the best fit to Gaussian peaks superimposed (black line). Each curve represents an average of 2–6 measurements.

The first derivative of the melting curves shown in Fig. 2 is shown in Fig. 3. As expected, the thermal melting data with the N-terminus alone measured by CD reveals no difference between experiments conducted in the presence or absence of ATM-3507 with peak values of 52.0 °C and 52.3 °C, respectively (Fig. 3a cf. d). However, both the C-terminus alone and in combination with the N-terminus showed significant increases in the peak values in the presence of ATM-3507. The peak value for the C-terminus was shifted from 44.4 °C to 46.5 °C (Fig. 3b cf. e) and for the combined peptides from 44.9 °C to 47.6 °C (Fig. 3c cf. f). We propose that ATM-3507 is able to increase the stability of the C-terminus of Tpm3.1 either alone or in the context of the overlap junction.

It is notable that the peak value for the peptide combination (44.9 °C) is very similar to the value of the C-terminal peptide (44.4 °C) and significantly lower than for the N-terminal peptide (52.3 °C). This suggests that the C-terminal peptide has a more dominant impact than the N-terminal peptide when present together in an overlap junction.

### Incorporation of ATM-3507 into the Tpm3.1/actin co-polymer

The CD spectroscopy thermal melts suggest that ATM-3507 may incorporate into the overlap junction and this is consistent with the observation that ATMs only nullify the ability of Tpm3.1 to protect actin filament stability when they are present during polymerization^19^. To test the integration of ATM-3507 into the Tpm3.1/actin co-polymer we generated a tritiated derivative of the compound. ^3^H-ATM-3507 was incubated with Tpm3.1 and then the mixture added to F-actin and polymerization allowed to proceed. After incubation for 1 h the mixture was centrifuged to collect the Tpm3.1/actin co-polymer and the amount of ^3^H-ATM-3507 incorporated was determined.

**Figure 4:**
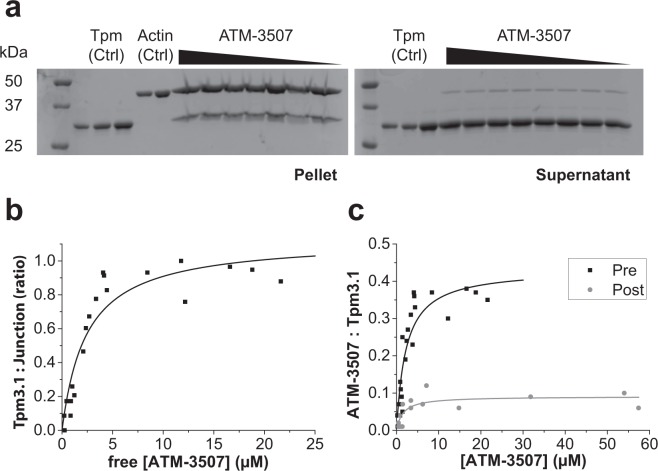
Binding and incorporation of ATM-3507 into Tpm3.1/actin filaments. (**a**) Increasing amounts of ^3^H-ATM-3507 (0–22 µM) pre-incubated with Tpm3.1 do not affect formation of Tpm-actin filament. Protein fractions of the Tpm-actin from both pellets and supernatants after centrifugation of the Tpm-actin mixture with increasing concentrations of ATM-3507. Full-length gels are presented in Supplementary Fig. 1. (**b**) The molar ratio of ATM-3507 to the junction formed by the N-terminal and C-terminal ends of Tpm dimers as a function of ATM-3507 concentration; n = 3. (**c**) Incorporation of ^3^H-ATM-3507 into Tpm/actin filament under two different conditions of (i) pre-incubated (Pre, black squares) ^3^H-ATM-3507 with 5 μM Tpm3.1 prior to actin addition, or (ii) post-incubated (Post, gray circles) ^3^H-ATM-3507 with the Tpm3.1/F-actin filaments; n = 4 for each curve.

As previously observed, increasing concentrations of ATM-3507 had no impact on the binding of Tpm3.1 to F-actin (Fig. 4a). ^3^H-ATM-3507 displayed concentration-dependent incorporation into Tpm3.1/actin co-polymers (Fig. 4b). The incorporation of ^3^H-ATM-3507 reached saturation at about 5 µM and had an apparent binding affinity of 2 µM. Calculation of the binding of ^3^H-ATM-3507 relative to Tpm3.1 overlap junctions present in the pellet suggested that at saturation the molar ratio approached one molecule of ^3^H-ATM-3507 per overlap junction.

Previous studies have suggested that ATM-3507 cannot impact the function of preformed Tpm3.1/actin co-polymers^19^. This was tested by repeating the binding experiment with the exception that ^3^H-ATM-3507 was incubated for an hour with preformed Tpm3.1/actin co-polymers. Only a low amount of binding was observed which was close to background (Fig. 4c). This confirms that optimal binding of ATM-3507 only occurs if the compound is present during polymerization.

### Structural studies of the ATM-3507-Tpm3.1 overlap junction

To better understand the effects of ATM-3507 binding on Tpm3.1 function, structural studies were undertaken. While the Tpm3.1 C-terminal peptide and the overlap complex of the two peptides in the presence or absence of ATM-3507 did not crystallize, the crystal structure of the N-terminal peptide of Tpm3.1 was solved at a resolution of 2.3 Å. The solved structure shows excellent geometry and good agreement with the x-ray diffraction data (Table S1). The refined structure contains four Tpm3.1 chains that form two coiled coils. This peptide is analogous to the previously solved Tpm1.1 N-terminal peptide structure^20^, and the structure is very similar to the Tpm1.1 coiled coil, with a root mean square deviation of 0.69 Å calculated with backbone atoms from residues 12-74 of the Tpm3.1 coiled coil. The structural similarity is perhaps surprising given that the Tpm3.1 N-terminus is coded by exon 1b, whereas the Tpm1.1 is coded by exon 1a which is shorter by 6 residues and only has 24% identity to Tpm3.1 at the N-terminus. On the other hand, comparison of the Tpm3.1 structure to the crystal structure of a non-muscle Tpm derived from the TPM1 gene^21^ (αTM1bZIP, a chimeric construct derived from the first 19 amino acids of tropomyosin fused with 18 amino acids of the leucine zipper from GCN4) shows some differences with a root mean square deviation of 1.71 Å calculated with alpha carbons from residues 12–35. One helix in the coiled coil aligns well, but the other shows significant variation. These differences could be a consequence of the different space groups between the crystals, the different isoforms used (55% sequence identity between the Tpm3.1 N-terminus and αTM1bZIP, not including the GCN4 residues), and the presence of leucine zipper protein GCN4 sequence in the αTM1bZIP construct. The first 6 (including the Ala-Ser and the N-terminal residues not expressed in Tpm1.1) and last 3 residues (including the engineered Cys) are not visible in the electron density maps and are most likely disordered. Similarly, these N-terminal residues were not visible in the αTM1bZIP crystal structure, so these residues may be disordered in all Tpms containing exon 1b.

**Figure 5:**
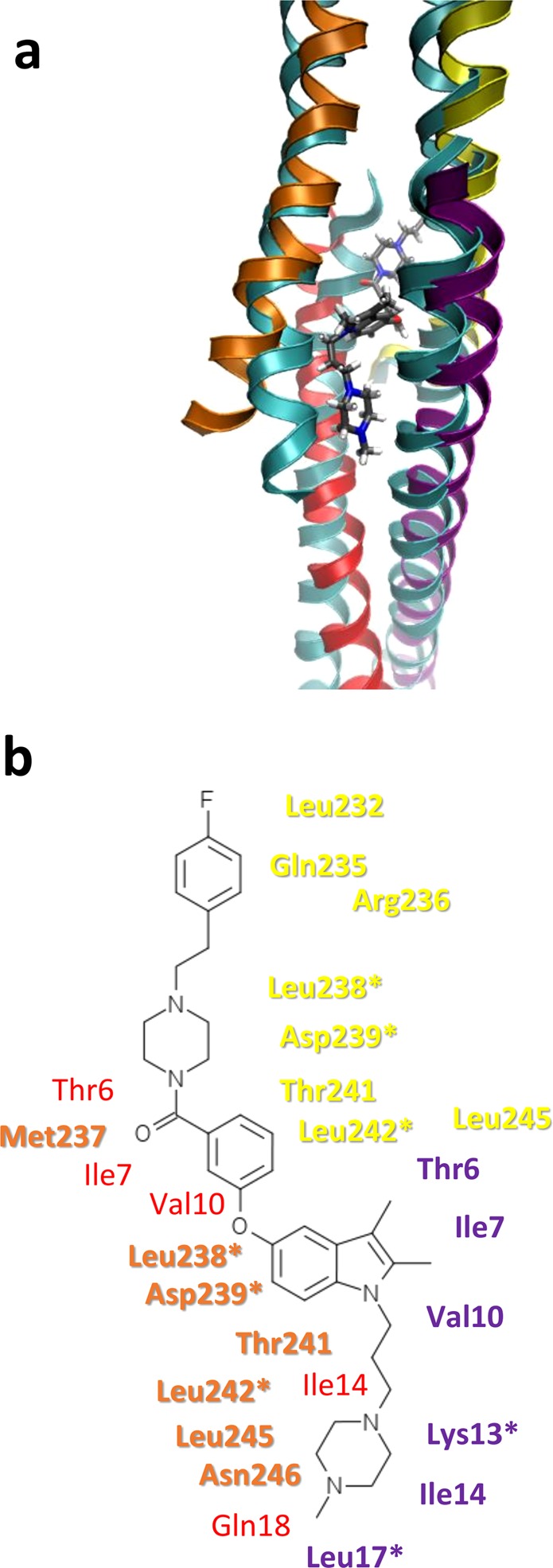
Binding of ATM-3507 as determined by molecular dynamics simulation of the docked Tpm3.1 overlap. (**a**) A representative structure of the Tpm3.1-ATM-3507 complex from MDS (the structure was built incorporating PDB structures 2G9J, 2K8X, and 6OTN and is shown with yellow and orange ribbons for the C-terminal peptides and red and purple ribbons for the N-terminal peptides). The ATM-3507 is shown in sticks with black carbons. The structure is superposed onto the unliganded Tpm3.1 model (cyan ribbons) for comparison. (**b**) A diagram of ATM-3507 with close protein contacts colored according to the ribbons in A shows the binding site consists of mostly hydrophobic residues that normally make up the center of the 4-helix bundle of the overlap. Residues with an asterisk are conserved in Tpm1.1.

Molecular dynamics simulations (MDS) were carried out on models of control Tpm3.1 N-/C-overlap junctions and also on this structure with ATM-3507 incorporated into its hydrophobic core. Note that molecular dynamics was restricted to the region of the overlap junctions, not to full-length Tpm3.1 or Tpm3.1 associated with F-actin, as structures of the latter have not been determined experimentally or been modelled. Both complexes were well-behaved over 30–40 ns production runs and snapshots from controls and ATM-3507 taken during MDS showed no evidence of distortion or complete separation of their component α-helices. Individual conformers during MDS were averaged and representative frames selected, showing that the ATM-3507 remains intimately associated with both C- and N-terminal Tpm3.1 chains in the overlap core, while the fluorophenyl and N-methylpiperazine groups project towards solvent (Fig. 5a). Contact maps between the liganded ATM-3507 and the drug shows numerous hydrophobic interactions with each of the four component chains and a few polar interactions toward the ends of ATM-3507 (Fig. 5b). No specific hydrogen bonding or charge interactions are noted between the protein and the compound, rather the binding site is dominated by non-specific interactions.

**Figure 6:**
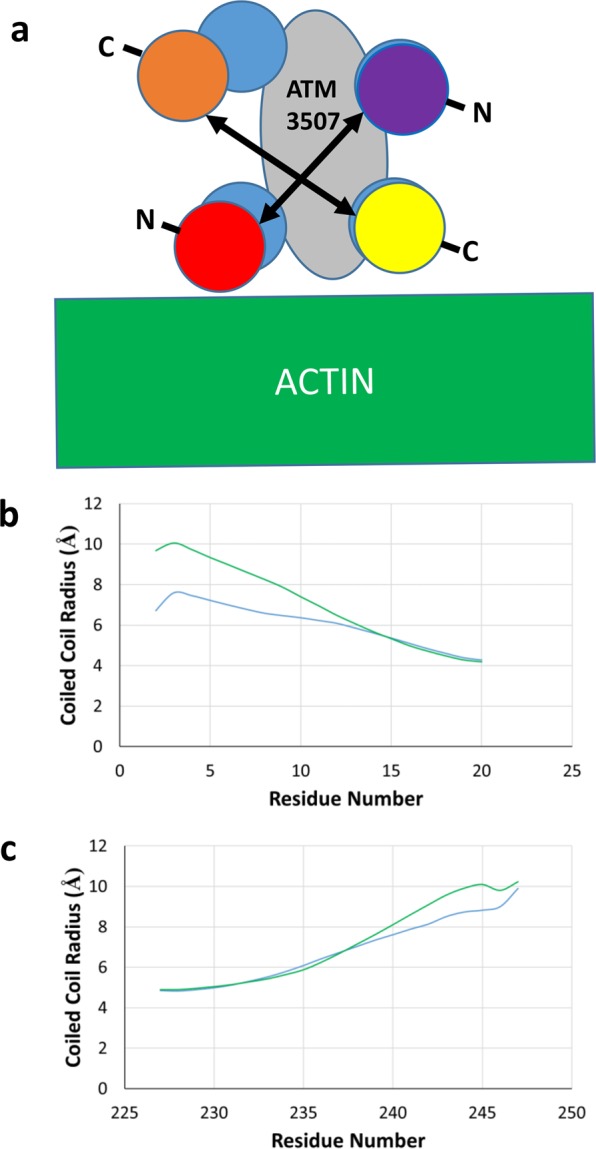
ATM-3507 causes a widening of the overlap helices. (**a**) Cartoon representation of the overlap junction viewed down the superhelical axis of Tpm where the helices are represented as circles colored as in Fig. 5 for the unliganded (cyan circles) and ATM-3057 complex (yellow and orange circles for the C-terminal peptides and red and purple circles for the N-terminal peptides) to show the approximate widening of the overlap (indicated by double headed arrows) from the compound (gray oval). The approximate position of actin is shown as determined by superposing the unliganded Tpm3.1 overlap model onto the cable model of Tpm1.1 and actin^22^. (**b**,**c**) The coiled coil radius was measured by Twister^40^, and the average radius over the last 10 ns of simulation is graphed per residue for the unliganded (cyan) and ATM-3507 complex (green). Note the increase in radius when ATM-3507 is included in the simulation.

Binding of ATM-3507 in the model appears to alter overlap geometry in several ways. First, during the MDS, the overlap is widened to accommodate the compound when compared to the unliganded model. In particular, one N-terminal and one C-terminal chain on one side of the overlap are moved outward (Fig. 6a). Further docking of the Tpm residues to models of the Tpm cable on F-actin^22^, suggests that the ATM drug may impose steric restrictions on typical regulatory translation of Tpm over the surface of actin^23^. The splaying can be quantitated using Twister (Fig. 6b) and shows about a 2 Å increase for the N-terminal chains with a lesser increase in the C-terminal chains.

**Figure 7:**
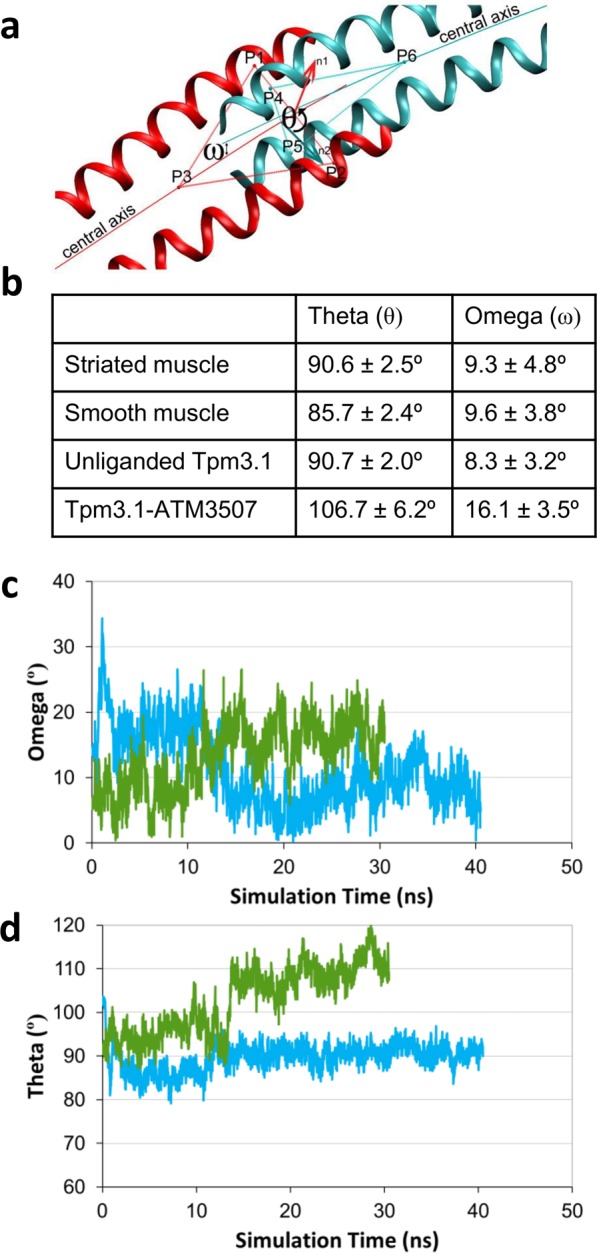
Analysis of overlap geometry from molecular dynamics simulations show ATM-3507 results in bending and twisting of the overlap. (**a**) The overlap twisting angle theta (θ) and bending angle omega (ω) were measured for the Tpm overlap using the previously published definitions^37^ (red ribbons are C-terminal chains and cyan ribbons are N-terminal chains). (**b**) θ and ω were measured for each frame of the 30–40 ns MDS and the average and standard deviations are shown. For comparison, the previously published values for skeletal muscle and smooth muscle Tpm are also tabulated^37^. The values suggest that the presence of the compound leads to a pronounced twisting and bending of the overlap. (**c**,**d**) The θ and ω angles are plotted as a function of simulation time for the unliganded Tpm3.1 overlap model (cyan) and the Tpm3.1-ATM-3507 complex simulation (green).

During MDS, twisting of overlap domain (θ) (relative to the domain’s central axis; see Fig. 7a) was increased about 15° in the drugged overlap construct when compared to the unliganded one, while on average its bending curvature (ω) was approximately 8° greater (Fig. 7b). Analysis of the angles over the course of the simulation (Fig. 7c,d) shows that the alteration in average angle occurs relatively quickly during the simulation and converges on a new, stable angle. Given the close packing and shape complementarity of ATM-3507 and the 4-helix bundle packing as well as the proximity of ATM-3507-core interactions, it is unlikely that the drug could insert into the 4-helix bundle once formed. Much more likely are interactions with splayed C- and N-terminal ends prior to the assembly of the overlap.

## Discussion

**Figure 8:**
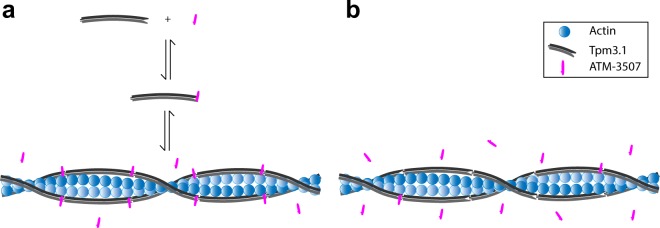
Schematic representation of ATM-3507 interactions with an actin filament. (**a**) pre-incubation with Tpm3.1 and (**b**) addition of ATM-3507 to an actin filament fully saturated with Tpm3.1 (post-incubation).

Previous studies have suggested that ATMs need to be present during Tpm3.1/actin co-polymerization to have *in vitro* activity^16,17,19^ and that these compounds have little impact on the binding of Tpm3.1 to F-actin *in vitro* and *in vivo*^19^. This study now provides molecular detail concerning the mechanism of ATM-3507 binding to Tpm3.1-containing actin filaments. ATM-3507 impact on the melting curve of the C-terminal, but not N-terminal, Tpm3.1 peptide suggests that ATM-3507 can bind directly to the C-terminus of Tpm3.1. This predicts an equilibrium between free and bound ATM-3507 with Tpm3.1 (Fig. 8). Furthermore, the findings that (i) ATM-3507 is incorporated into the Tpm3.1/actin co-polymer, (ii) ATM-3507 reaches maximal binding at approximately one molecule per Tpm3.1 junction, (iii) ATM-3507 can interact with the 4-helix overlap created by the interaction of N- and C-termini and (iv) MDS predicts that ATM-3507 is inserted in a pocket created by the 4-helix overlap junction predict a model in which ATM-3507 bound to the C-terminal of Tpm3.1 is incorporated into the overlap junction during polymerization (Fig. 8).

The model shown in Fig. 8 is a combination of a conventional equilibrium of ATM-3507 binding to Tpm3.1 and a non-equilibrium relationship in which the ATM-3507 becomes ‘locked’ in place. The MDS predicts that once bounded by the 4-helix overlap, it is unlikely that ATM-3507 could be in a conventional equilibrium with an unbound pool of ATM-3507. Thus, the binding affinity of approximately 2 µM derived from Fig. 4b does not represent a true equilibrium.

The recent demonstration that free Tpm3.1 dimers can exchange with Tpm3.1 dimers present in a Tpm3.1/actin co-polymer independent of filament turnover^24,25^ provides a potential alternative mechanism of delivery of ATM-3507 into a preexisting filament. If this occurs, it does not appear to be rapid in this cell-free assay because incubation of preformed Tpm3.1/actin filaments in the presence of free Tpm3.1 and ATM-3507 results in only low levels of incorporation of ATM-3507. It therefore appears most likely that the primary mechanism of incorporation of ATM-3507 into Tpm3.1/actin filaments is via delivery of the compound bound to Tpm3.1 dimers during polymerization in this cell-free assay. However, we cannot rule out this alternative mechanism of drug incorporation in the more complex regulatory environment of the cell.

Many ATMs have been developed and three (with very different structures), have been studied in a range of cell and animal experiments^16–18^. Models of the binding of these molecules to the C-terminus of Tpm3.1 reveal a diversity of interactions with C-terminal side chains^26^. The MDS modelling suggests even more extensive variation in side chain interactions become possible in the 4-helix overlap. This suggests that there is substantial flexibility in the chemical options available for interaction with the C-terminus and the 4-helix overlap that may be intrinsic to drugs that interact with coiled coils. In this way, coiled coils may present a very different opportunity for drug design in comparison with more conventional drug binding ‘pockets’ on the surface of target proteins.

The integration of ATM-3507 into the Tpm3.1/actin co-polymer, most likely into the overlap region, suggests that it should be possible to target the different Tpm isoforms. The greatest differences between the Tpm isoforms, apart from the alternatively spliced exon 6, lie in the highly conserved N- and C-termini that form the overlap junction^9^. The insight provided by the MDS analysis identifies the opportunities to design compounds that exploit these sequence differences to have isoform preferred integration into the 4-helix overlap. The development of such compounds has the potential to bring a chemical biology approach to understanding the mechanisms of Tpm isoform-specific function.

Chemical biology brings a new way of thinking about the dissection of biological mechanisms. The integration of ATM-3507 into the Tpm3.1/actin co-polymer provides the opportunity to test the role of the overlap junction in the biology of Tpm. Tpm3.1/actin co-polymers interact with many actin binding proteins and myosin motors and it seems unlikely that all ATMs will equally impact all these interactions, as variations in molecular size, shape, and orientation in the binding site would be expected to alter the geometry of the overlap junction in different ways. This is particularly relevant in terms of the impact of compounds on the lateral movement of Tpm3.1 across the surface of actin, where such changes may not uniformly affect all filament interactions. Structural analogues of ATM-3507 therefore have the potential to selectively impact only a subset of Tpm3.1 functions, providing valuable tools to study the role of Tpm as a regulator of actin filament function.

## Materials and Methods

### Chemistry

ATM-3507 was prepared as described previously^27^. ^3^H-ATM-3507 (specific activity: 25 Ci/mmol) was purchased from American Radiolabeled Chemicals Inc. (St. Louis, MO) via BioScientific Pty. Ltd. (Sydney, Australia) as a 2.5 mM solution in DMSO.

### Design and cloning of C- and N-terminal Tpm3.1 constructs

The cDNA and amino acid sequences of N_82AA_Tpm3.1 and C_109AA_Tpm3.1 are shown in Supplementary Materials Tables S2 and S3, respectively. The human Tpm3.1 cDNA sequence (NP_705935.1) was used as a template to generate both peptides. The N-terminal peptide contains an Ala-Ser extension that mimics acetylation^28^ of the native Tpm3.1^6^, followed by the first 80 amino acids with minor modifications [change of Ala80 to Cys; introduction of STOP codon (TAG) to residue 81]. The sequence was optimized for expression in *E. coli* using the IDT codon optimization tool (https://sg.idtdna.com/CodonOpt), synthesized (GenScript Biotech Corp., NJ, USA) and subcloned into pET-26b(+) vector using EcoRI/HindIII restriction sites. The C-terminal peptide is based on Nitanai *et al*.^29^ and contains the initial 25 residues of the GCN4 leucine-zipper as the stabilizing alpha-helical N-terminal extension, followed by the last 109 AA residues. The sequence was generated and subcloned into pET-26b(+) vector via Sall and Hindlll restriction sites (GenScript Biotech Corp., NJ, USA).

### Protein expression and purification

Tpm N_82AA_Tpm3.1 and C_109AA_Tpm3.1 plasmid-containing bacteria were grown on LB/Knm agar plates (100 μg/mL) O/N at 37 °C. 20 mL LB/Knm broth (100 μg/mL) was inoculated with one colony and grown O/N at 37 °C. The O/N culture was used to inoculate 3 L (5 × 0.6L) LB/Knm media and grown at 37 °C until OD600 ~0.6. Cells were induced by the addition of 1 mM isopropylthio-β-D galactosidase (IPTG, Gold Biotechnology), grown for 3 h and centrifuged [8,280 *g*, 15 min (SLA-3000 rotor, Thermo Scientific)]. The pellet was washed with PBS, centrifuged and stored at −40 °C. The pellet was resuspended in 45 mL ice-cold lysis buffer (0.5 M NaCl, 20 mM Tris pH 7.5, 5 mM MgCl_2_, 1 mM EGTA, 1 mM NaN_3_), and one protease inhibitor tablet (Roche) and PMSF (1 mM; Sigma Aldrich) added. Cells were lysed on ice by sonication (6 cycles, 30 sec on/off, amplitude 40%, 90% duty cycle) and quickly transferred to an 80 °C water bath for 10 min. Contaminant proteins are irreversibly denatured while heat stable Tpm refolds after cooling. The sample was transferred into a pre-cooled centrifugation tube, gently mixed and left at −20 °C for 8 min, followed by centrifugation [47,810 *g*, 4 °C, 45 min (SS-34 rotor, Sorvall RC6C, Thermo Scientific)]. Supernatant pH was lowered to 4.7, centrifuged [3900 *g*, 4 °C, 15 min (SX 4250 rotor, Alegra X-22 R, Beckman Coulter)] and pellet re-suspended (50 mM NaCl, 2 mM Tris, 1 mM DTT, 1 mM azide, pH 7.5). This step was repeated. Re-suspended pellet was filtered (0.22 μm) and purified in 3 steps. (i) Anion exchange chromatography: Protein solution was loaded on 2 × 5 mL HiTrap HQ columns (GE Healthcare) equilibrated with 10 CV of buffer A (50 mM NaCl, 2 mM Tris pH 7.5, 2 mM DTT, 1 mM NaN_3_). Peptides were eluted by a linear gradient (15 to 65%) of buffer B (1 M NaCl, 2 mM Tris pH 7.5, 2 mM DTT, 1 mM NaN_3_) over 10 CV. Protein peak fractions were analysed by SDS-PAGE, pooled and dialysed against CHT1 buffer (1 M NaCl, 10 mM sodium phosphate pH 7.0, 0.5 mM DTT) O/N. (ii) Cation exchange chromatography: Protein solution was loaded on a ceramic hydroxyapatite column (CHT type I, 5 mL cartridge, BioRad) equilibrated with 10 CV of CHT1 buffer. Sample was eluted by a linear gradient (10 to 160 mM) of sodium phosphate over 30 CV. Protein peak fractions were analyzed by SDS-PAGE, pooled and concentrated using Amicon Ultra-15 centrifugal filters (Merc Millipore). (iii) Protein peak fractions were analysed by SDS-PAGE, pooled and dialysed against a buffer solution containing storage buffer (20 mM Tris pH 7, 100 mM KCl, 5 mM MgCl_2_, 1 mM NaN_3_, 0.5 mM DTT). Purified proteins were run on a size exclusion chromatography column (HiLoad 16/600vSuperdex 75 pg; GE Healthcare) using the storage buffer. Protein (up to 20 mg/mL) was snap frozen in liquid nitrogen and stored at −80 °C.

### Circular dichroism

CD spectra and thermal unfolding isotherms of N_82AA_Tpm3.1 and C_109AA_Tpm3.1 peptides were acquired on a Chirascan Plus CD Spectrometer (Applied Photophysics Limited, U.K.) in stoppered 0.5 mm cuvettes (48/Q/0.5; Starna Scientific Ltd.). Proteins were dialyzed O/N against 10 mM NaH_2_ PO_4_, 150 mM NaCl, 75 µM TCEP (added prior to experiment), 1% (v/v) acetonitrile pH7 buffer. Final concentration of peptides was 20 µM. Tpm samples containing ATM-3507 (100 µM) were incubated at 37 °C O/N. CD spectra were measured from 195 to 260 nm with a 1 nm step size and a 1.0 mm bandwidth, taking 3 to 4 averages at 37 °C. The thermal unfolding was carried out at 222 nm over the range 20–65 °C at the rate of 1 °C/min. Sample was cooled down to the initial temperature and heating procedure was repeated 2 to 3 times. To obtain information of the major thermal transitions from individual isotherms, data were further smoothed by the Savitzky-Golay method (5 point window), differentiated and then fitted to multiple Gausian peaks as described previously^30^.

### Radioligand binding assay

The affinity of ATM-3507 for F-actin/Tpm3.1 was measured by co-sedimentation according to a modification of Bonello *et al*.^19^ ^3^H-ATM-3507 was purchased from American Radiolabeled Chemicals, Inc (St. Louis, MO), with a specific activity of 25 Ci/mmol and a purity of above 98%. It was diluted with cold ATM-3507 to a specific activity of 125 mCi/mmol.

For the pre-incubation assay, serial dilutions of ^3^H-ATM-3507 (64 to 0.25 µM) were incubated with Tpm3.1 at 5 µM for 10 min in buffer A (10 mM Tris-HCl pH 7.5, 100 mM NaCl, 2 mM MgCl_2_, 1 mM EGTA, 0.5 mM DTT). Samples were mixed with F-actin (Hypermol EK, Bielefeld, Germany, #8101-03) at a fixed concentration of 9 μM and incubated for 1 h at RT. Samples containing actin-Tpm filaments with increasing ATM-3507 concentrations (25 µL final volume) were centrifuged at 3,000 *g* (Eppendorf 5415D) for 20 min, 25 °C. Supernatant fractions were transferred into HIMAC ultracentrifuge tubes (339133A). 1 µL of supernatant was measured to calculate the concentration of ^3^H-ATM-3507 in buffer. Samples were centrifuged [100,000 *g*, 20 min, RT (Hitachi Micro-Ultracentrifuge, S100-AT3 Rotor)] to pellet F-actin and associated Tpm. The pellet was carefully washed 3 times with 100 µL buffer A containing 0.2 mM ATP. Amount of ^3^H-ATM-3507 in Tpm3.1/actin filament pellets was determined by β-scintillation counter (TriCarb® liquid scintillation analyzer; Perkin Elmer, Inc.).

Post-incubation assay was conducted according to the pre-incubation binding assay except F-actin and Tpm3.1 were mixed together to form the F-actin/Tpm3.1 filament prior to addition of ^3^H-ATM-3507. The concentration of free ATM-3507 in the supernatant was calculated from the specific activity of ^3^H-ATM-3507 in the supernatant. ^3^H-ATM-3507:Tpm was calculated from the specific activity of ^3^H-ATM-3507 (β-scintillation counter) in the pellet fraction. The experimental data were fitted to a non-linear regression model (one site-specific binding) in GraphPad Prism 7.04.

### Co-sedimentation assay

Co-sedimentation was conducted according to the pre-incubation binding assay described above with modifications. Increasing concentrations of ^3^H-ATM-3507 (0.25–64 µM) were incubated with 5 μM Tpm3.1 for 10 min (50 µL final volume) in buffer A (10 mM Tris-HCl pH 7.5, 100 mM NaCl, 2 mM MgCl2, 1 mM EGTA and 0.5 mM DTT) prior to mixing with F-actin (9 μM) and then incubated for another 1 h at RT. The samples were centrifuged [435,680 *g*, 30 min, RT (Hitachi Micro-Ultracentrifuge, S100-AT3 Rotor)] to pellet F-actin and associated Tpm. Pelleted fractions were then carefully washed 3 times with 100 µL buffer A containing 0.2 mM ATP. Pellet and supernatant fractions were run on SDS-PAGE gel and visualized by Coomassie blue staining (Gel Doc™ EZ). Protein bands were quantified by densitometry (Epson Perfection V750 Pro scanner and ImageJ). The ratio of the Tpm/actin density was plotted as a function of the concentration of free Tpm in the supernatant.

### Crystal structure of N_82AA_Tpm3.1, homology modeling, and molecular dynamics

Crystals of N_82AA_Tpm3.1 were grown in hanging drops by mixing purified N_82AA_Tpm3.1 in storage buffer at 20 mg/ml with an equal volume of reservoir solution (0.1M BisTris pH 6.5, 0.2M Li_2_SO_4_, 12% polyethylene glycol 3350) on a siliconized cover glass and equilibrating over reservoir solution at 20 °C. Crystals were incubated in reservoir solution supplemented with 23% (v/v) glycerol and flash frozen in liquid nitrogen for x-ray data collection. Data were collected at NSLS-II beamline 17-ID using x-rays with a wavelength of 0.979338 Å. Data were indexed and integrated with XDS^31^. Space group determination and merging were performed using the programs POINTLESS and AIMLESS^32^. The structure was solved by molecular replacement using the program Phaser^33^ using the Tpm1.1 N-terminal piece crystal structure^20^; (PDB code 1IC2) as a search model. The model was subsequently built through iterative stages of refinement and manual rebuilding using the programs Phenix^33^ and Coot^34^, respectively. For the temperature factors, TLS parameters were refined in the final structure with each protein chain refined as a single TLS group. The final model consists of four Tpm chains, waters, and solvent atoms. Refinement and data collection statistics are shown in Table S1.

A homology model of Tpm3.1 was constructed using the methods outlined for construction of the low molecular weight Tpm model^35^, but utilizing the Tpm3.1 amino acid sequence and the N_82AA_Tpm3.1 crystal structure. Briefly, the N-terminal NMR structure of a non-muscle Tpm (PDB code 2K8X^35^) was used to replace the N-terminal portion in the NMR model^7^ of the striated muscle overlap (PDB code 2G9J^7^) by superposition of residues 6–34 of the former on residues 1–29 of the latter. This backbone was then used to thread the Tpm3.1 sequence onto the N- and C-terminal fragments, creating the initial overlap model. The model was extended out to residues 1–77 and 169–248 using the crystal structure coordinates for residues 20–77 and a standard coiled-coil structure for residues 169–228. These models were then subjected to molecular dynamics simulations using the program NAMD^36^ in explicit solvent as previously described for striated muscle Tpm overlap structures^37^. Briefly, the structure was placed in a solvent box using the solvate plugin in VMD^38^ with a cushion of 15 Å and a boundary of 1.4 Å. NaCl was added to a final concentration of 0.15M with the ionize plugin. Solvent ions were minimized first in NAMD, then constrained minimization of the whole system was undertaken with gradual release of the constraints. The system was then heated to 300 K with constrained protein and constant volume. At 300 K, the pressure was maintained at 1 bar using a Langevin piston and the constraints were gradually released prior to production runs. Calculation of the bending angle ω (omega) and twisting angle θ (theta) of the overlap were calculated as defined previously^37^.

Docked structures of ATM-3507 to the C-terminal portion of the model were as published^17^. The docked ATM-3507/C-terminal domain coordinates were then superimposed on the Tpm3.1 homology model C-terminal chains from the MD simulation to generate a starting model for MDS of the ATM-3507 complex, which was carried out similarly to the wild-type. Parameter and topology files for the ATM-3507 compound were generated using the program MATCH^39^.

### Accession numbers

Coordinates and structure factors have been deposited in the Protein Data Bank with the accession code 6OTN. Authors will release the atomic coordinates and experimental data upon article publication.

## Supplementary information

Supplementary Material
