# Supplementary material for: Molecular integration of the anti-tropomyosin compound ATM-3507 into the coiled coil overlap region of the cancer-associated Tpm3.1
Source: Sci Rep. 2019 Aug 2;9:11262. doi: 10.1038/s41598-019-47592-9 (PMC6677793; doi:10.1038/s41598-019-47592-9)
Supplement: Supplementary file 1 — Supplementary Material [file 41598_2019_47592_MOESM1_ESM.pdf]

Supplementary information

Molecular integration of the anti-tropomyosin compound ATM-3507 into the coiled coil overlap region of the cancer-associated Tpm3.1

Miro Janco[a, ‡], Michael J. Rynkiewicz[b, ‡], Liang Li[a, ‡], Jeff Hook[a], Eleanor Eiffe[a], Anita Ghosh[b], Till Böcking[c], William J. Lehman[b], Edna C. Hardeman[a], Peter W. Gunning[a,#]

[a] School of Medical Sciences, University of New South Wales Sydney, Sydney, NSW 2052, Australia

[b] Department of Physiology & Biophysics, Boston University School of Medicine, 72 East Concord Street, Boston, MA 02118, USA

[c] Single Molecule Science and ARC Centre of Excellence in Advanced Molecular Imaging, University of New South Wales Sydney, Sydney, NSW 2052, Australia

Corresponding author:
# Email: p.gunning@unsw.edu.au

Fig. S1. Full length SDS gels used as an example of the co-sedimentation assay in the Fig. 4a.

a

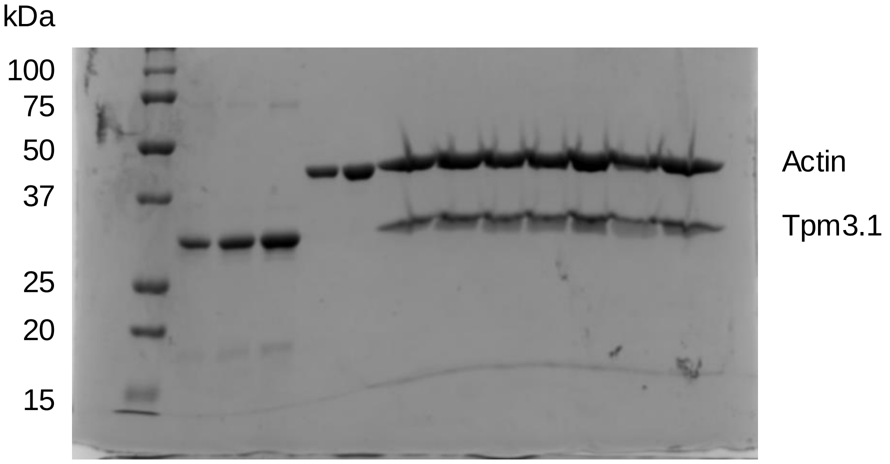

b

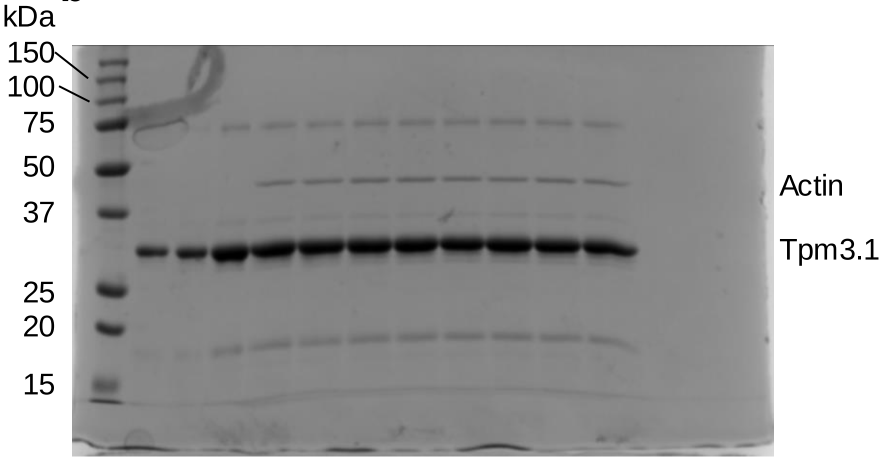

Table S1. Crystallographic data collection and refinement statistics for N_82AA_Tpm3.1.

PDB Code	6OTN
**Data Collection**	
X-Ray Source	NSLS II beamline 17ID-AMX
Detector	Eiger9M pixel-array
Wavelength (Å)	0.979338
Resolution, last shell (Å)	29.5-2.4 (2.46-2.4)
Space group	$P\ 4_3\ 2_1\ 2$
Unit cell, a, b, c (Å)	a = b = 97.201, c = 93.100
Unit cell, α, β, γ (°)	α = β = γ = 90
No. of Reflections	274283
No. of Unique Reflections	17984
Redundancy (last shell)	15.3 (14.4)
Mean I/σ (last shell)	23.2 (3.4)
Completeness (last shell)	99.3 (90.9)
R-meas (last shell)	0.087 (0.824)
R-pim (last shell)	0.030 (0.286)
Wilson B-factor	48.34
Average Mosaicity (°)	0.09
**Refinement**	
Working Set (last shell)	17592 (1049)
Test Set (last shell)	1765 (107)
R_{work} (last shell)	0.2348 (0.2987)
R_{free} (last shell)	0.2658 (0.3482)
RMSD of Bond Lengths (Å)	0.002
RMSD of Bond Angles (°)	0.460
Total Number of Atoms	2388
Protein Residues	282
Protein	2311
Sulfate	5
Water	72
Average B-factor	
Protein	72.10
Sulfate	107.92
Water	53.49
Ramachandran Favored, Allowed, Outliers (%) from MolProbity	100, 0, 0

Table S2. cDNA and amino acid sequence of N_82AA_Tpm3.1 construct.

**cDNA sequence of human N_82AA_Tpm3.1** (optimized for *E. coli*)<br><br>GST AGT GCT GGG ATT ACC ACT ATT GAA GCA GTT AAA CGT AAA ATT CAA GTG<br>TTA CAG CAA CAG GCT GAT GAC GCA GAG GAG CGC GCT GAA CGC TTA CAA CGC<br>GAG GTA GAA GGG GAA CGC CGC GCG CGC GAG CAA GCA GAA GCG GAA GTG<br>GCG TCG CTT AAT CGC CGT ATC CAG TTA GTC GAA GAA GAA CTT GAC CGT GCC<br>CAA GAA CGC TTG GCC ACC GCG TTA CAG AAG TTA GAG GAA TGT –Stop (TAG)
**Amino acid sequence of human N_82AA_Tpm3.1**<br><br>Initial sequence selected:<br>ASAGITTIEAVKRKIQVLQQQADDAEERAERLQREVEGERRAREQAEAEVASLNRRIQLV<br>EEELDRAQERLATALQKLEEAE<br><br>Final sequence:<br>ASAGITTIEAVKRKIQVLQQQADDAEERAERLQREVEGERRAREQAEAEVASLNRRIQLV<br>EEELDRAQERLATALQKLEEC-Stop

Table S3. cDNA and amino acid sequence of C_109AA_Tpm3.1 construct.

**cDNA sequence of human C_109AA_Tpm3.1** (optimized for *E. coli*)
**ATG GAT AAA GTG GAA GAG CTG TTG AGC AAA AAC TAT CAT CTG GAA AAC GAA GTG GCG CGT CTG AAA AAG CTG GTG** CTT GAG CGT ACC GAG GAG CGC GCC GAG CTT GCT GAA AGT CGT TGT CGT GAG ATG GAT GAG CAG ATT CGT CTT ATG GAC CAG AAC TTG AAA TGC TTA TCG GCT GCT GAG GAG AAG TAC TCT CAG AAA GAG GAT AAA TAT GAA GAA GAA ATT AAA ATT TTG ACG GAT AAA CTG AAA GAG GCA GAG ACC CGT GCC GAA TTC GCT GAG CGT TCT GTG GCC AAG TTA GAA AAG ACT ATC GAT GAT CTT GAA GAC AAA CTG AAG TGT ACA AAG GAA GAG CAT CTT TGC ACC CAG CGT ATG TTG GAC CAG ACT TTG TTA GAC CTT AAT GAA ATG
**Amino acid sequence of human C_109AA-Tpm3.1**
**MDKVEELLSKNYHLENEVARLKKLV**LERTEERAELAESRCREMDEQIRLMDQNLKCLSAAEEKYSQKEDKYEEEIKILTDKLKEAETRAEFAERSVAKLEKTIDDLEDKLKCTKEEHLCTQRMLDQTLLDLNEM

Note The construct contains 25 AA residues leucine-zipper sequence (bold) of GCN4 as an N-terminal extension.
